# The Gap in Mental Health Service Utilization Among United Methodist Clergy with Anxiety and Depressive Symptoms

**DOI:** 10.1007/s10943-022-01699-y

**Published:** 2022-12-12

**Authors:** Blen Biru, Jia Yao, James Plunket, Celia F. Hybels, Eunsoo Timothy Kim, David E. Eagle, Jessica Y. Choi, Rae Jean Proeschold-Bell

**Affiliations:** 1 Center for Health Policy and Inequalities Research, Duke Global Health Institute, Duke University, 310 Trent Drive, Durham, NC 27705 USA; 2 Department of Psychiatry and Behavioral Sciences, Center for the Study of Aging and Human Development, Duke University Medical Center, Durham, NC 27705 USA

**Keywords:** Mental health services, Depressive symptoms, Anxiety symptoms, Emotional distress, Clergy

## Abstract

Clergy are tasked with multiple interpersonal administrative, organizational, and religious responsibilities, such as preaching, teaching, counseling, administering sacraments, developing lay leader skills, and providing leadership and vision for the congregation and community. The high expectations and demands placed on them put them at an increased risk for mental distress such as depression and anxiety. Little is known about whether and how clergy, helpers themselves, receive care when they experience mental distress. All active United Methodist Church (UMC) clergy in North Carolina were recruited to take a survey in 2019 comprising validated depression and anxiety screeners and questions about mental health service utilization. Bivariate and Poisson regression analyses were conducted on the subset of participants with elevated depressive and anxiety symptoms to determine the extent of mental health service use during four different timeframes and the relationship between service use and sociodemographic variables. A total of 1,489 clergy participated. Of the 222 (15%) who had elevated anxiety or depressive symptoms or both, 49.1% had not ever or recently (in the past two years) seen a mental health professional. Participants were more likely to report using services currently or recently (in the past two years) if they were younger, had depression before age 21, or "very often" felt loved and cared for by their congregation. The rate of mental health service use among UMC clergy is comparable to the national average of service use by US adults with mental distress. However, it is concerning that 49% of clergy with elevated symptoms were not engaged in care. This study points to clergy subgroups to target for an increase in mental health service use. Strategies to support clergy and minimize mental health stigma are needed.

## Introduction

Clergy conduct work that is challenging due to its interpersonal nature, multiple responsibilities, and emotional content. Clergy work includes navigating competing demands, such as consoling others during difficult times, crisis intervention, preaching, performing administrative responsibilities, and leading activities for which they may not have had training (e.g., fundraising) (Bledsoe et al., 2013; Proeschold-Bell et al., 2013). On top of these external demands, many clergy experience internal struggles—such as feeling that they could do more or doubting their calling in life (Piper, 2015). While clergy generally report high levels of satisfaction with their work (Smith, 2007), the combination of these demanding activities and high internal and external expectations creates considerable stress and places clergy at risk for mental and occupational distress, including experiencing depression and anxiety (Frenk et al., 2013; Terry & Cunningham, 2020).

Several studies suggest above-average depressive symptoms among certain groups of clergy. United Methodist Church (UMC) clergy in North Carolina experience higher rates of elevated depressive symptoms compared to data on US adults using the same depression screener; although there was not a good national comparison, the study authors also found high rates of anxiety symptoms in clergy (Proeschold-Bell et al., 2013). Drawing on a nationally representative sample of UMC clergy, findings from the 2021 UMC Clergy Well-Being Survey also found a high rate of elevated depression symptoms, with 14% of clergy having scores indicating moderate or higher depressive symptoms (Wespath Center for Health, 2021). Studies of Roman Catholic secular clergy also suggest concerns about depression symptoms (Knox et al., 2002).

Despite the need to address clergy mental distress, less is known about whether clergy, helpers themselves, seek help to address their mental health problems and from whom they receive help. Research has shown stigma toward mental illness being associated with lower mental health treatment seeking behavior and poorer health outcomes (Shrivastava et al., 2012). Clergy may fear being stigmatized for engaging in mental health therapy, possibly because seeking mental health support may suggest they are unfit to lead their congregation or show a lack of faith (Meek et al., 2003; Proeschold-Bell et al., 2013). Alternatively, clergy might be highly aware of mental health treatment needs and likely recognize the benefits of mental health treatment for themselves*.* In a national survey in the USA, 25% of respondents with mental illness reported seeking help first from a clergy member; this figure was higher than the percentage of respondents seeking help first from psychiatrists (16.7%) or general physicians (also 16.7%), indicating that clergy are on the front lines of mental health care and are trusted by parishioners (Bledsoe et al., 2013; Wang et al., 2003).

In the current study, we investigated mental health service utilization among UMC clergy in North Carolina, drawing on survey data with a large sample size and a high response rate. Response rates are particularly important when studying mental health, because participants experiencing distress may be less likely to respond (e.g., due to less energy or interest) Center for Behavioral Health Statistics and Quality (2017). This study is important as it assesses whether clergy get the treatment they can benefit from and identifies potential gaps in care for certain subsets of clergy. Attending to the mental health of clergy will not only help them as individuals, but will also help the health and well-being of congregants they serve, as a leader’s health can impact those in the space they occupy (Little et al., 2007; Terry & Cunningham, 2020). Furthermore, clergy’s actions of seeking mental health care when needed might serve as an example and influence the actions of people who see them as role models (Anshel & Smith, 2014; Boyatzis et al., 2011).

## Methods

### Data Collection

This study draws on data from the Clergy Health Initiative Panel Survey, a longitudinal study of all the United Methodist Church (UMC) clergy in North Carolina (NC) from 2008 to the present. The sample for the current analyses comes from the 2019 survey wave, which is the only wave to include many of the survey items used in these analyses. The inclusion criteria for the 2019 survey were all clergy in the NC and Western NC UMC Annual Conferences (all full- and part-time appointed church pastors, district superintendents, deacons, bishops, extension ministers, previously appointed but currently disabled clergy, and clergy fully retired for fewer than four years). The survey was self-administered online, took on average 60 min, and included items related to mental, physical, and spiritual well-being. The research was approved by the Duke University Campus Institutional Review Board under protocol 2017–1197. Informed consent was obtained from all participants. The 2019 self-administered survey yielded a 73% response rate (*N* = 1,489).

We report on the subsample of participants with elevated depressive and anxiety symptoms according to the 2019 survey. For the analyses, we included only participants who experienced elevated anxiety and/or depressive symptoms within the past two weeks of completing the 2019 survey in order to assess the mental health treatment utilization among the subgroup that might most benefit from treatment.

### Measures

#### Mental Distress

Depressive symptoms were measured using the well-validated Patient Health Questionnaire-9 (PHQ-9), a nine-item scale with scores ranging 0–27. The scale allows participants to report the frequency of specific depressive symptoms within the past 2 weeks on a 0–3 scale (not at all = 0, several days = 1, more than half the days = 2, nearly every day = 3). Sum scores of 10 and above indicate elevated depressive symptoms (Kroenke et al., 2001). Anxiety symptoms were measured using the Generalized Anxiety Disorder-7 (GAD-7)—a seven item scale with scores ranging from 0 to 21. Participants reported the frequency of their experience related to each of the seven anxiety screening questions over the last 2 weeks on a 0–3 scale (with the same response options as in the PHQ-9). Sum scores of 8 and above indicate elevated anxiety symptoms (Spitzer et al., 2006).

#### Mental Health Service Utilization Timeframe

Mental health service utilization was measured using the following three survey items:

- *Have you ever in your life seen a mental health professional for treatment of depression, anxiety, or stress?*
- *Are you currently seeing a mental health professional to improve or maintain your mental health, including for depression, anxiety, or stress?*
- *In the past 2 years, have you seen a mental health professional to improve or maintain your mental health, including for depression, anxiety, or stress?*

From these items, we constructed three outcome measures for this study, each based on mental health service use during specific timeframes: 1) current vs in the past or never; 2) current or within two years vs more than two years ago or never; and 3) ever vs. never.

#### Perceived Congregant Support

For an exploratory analysis, we included the four-item Religious Support Short Form (Krause, 1999). The items measure emotional support received from congregants, emotional support given to congregants, negative interactions with congregants, and anticipated support from congregants (e.g., “How often do the people in your congregation make you feel loved and cared for?” Response options were “very often”, “fairly often”, “once in a while” and “never”).

### Analysis

We first described the characteristics of both the overall (parent) sample and the analysis sample of those with current anxiety and/or depressive symptoms. We then described the timeframe of mental health service utilization in four mutually exclusive categories (current, not current but in the past two years, more than two years ago, and never) by participants’ current mental distress status, reporting counts and percentages. We examined the bivariate relationship between the timeframe and mental distress status through a Chi-square test.

Prior research has found associations establishing a relationship between mental health-seeking behavior and several demographic characteristics, which we therefore included in the analyses: gender, marital status, age, education level, rural residence, insurance status, financial stress, social isolation, and depression before age 21 (Kansiewiez et al., 2022; Magaard et al., 2017). We included rural residence because rural areas often have proportionally fewer mental health providers (Andrilla et al., 2018). We included years of experience in ministry because occupation duration could potentially relate to risk of mental distress and treatment seeking behavior, and because having established relationships from longer occupation duration has been found to be associated with clergy burnout (Jackson-Jordan, 2013). We reported counts and percentages of the aforementioned sociodemographic characteristics except for experience in ministry and social isolation because they were not found to be significantly associated with mental health service utilization in bivariate analysis. Chi-square tests were used to assess the bivariate relationships between the sociodemographic characteristics and mental health service utilization timeframe, both in the four categories described in the Measures section and as a dichotomized variable (as in Table 2).

We then described the types of mental health professionals and non-mental health professionals that participants reported going to for improving or maintaining their mental health. Counts and percentages were reported separately for participants who were currently receiving care vs had received mental health care in the past two years. We described perceived congregant support by reporting counts and percentages for each item from the Religious Support Scale. We examined, as a bivariate analysis, whether each item was significantly associated with the mental health service utilization timeframe, both in the four categories and as a dichotomized variable (as in Table 4), using Chi-square tests. One religious support item (“*How often do the people in your congregation make you feel loved and cared for?”*) that was significantly associated with the four mental health service utilization timeframes was selected as an independent variable for multivariable modeling.

Finally, we used multivariable modified Poisson regression to model each of three binary mental health service utilization timeframe outcomes named in the Measures. For each of the continuous covariates (age and financial stress), we ran model specifications including its square term to test whether its relationship with the outcome is nonlinear, and then include the squared term for the final model if a statistically significant association, or a trend toward significance, was found between the square term and the outcome. All analyses were conducted using STATA version 16.1 (StataCorp LLC, College Station, TX, USA, 2021).

## Results

A total of 1,489 participants completed the 2019 survey; 64% were male and 91% were White. Of the full sample, 222 participants (15%) had scores on the anxiety and/or depressive symptoms measures that indicated elevated symptoms in the last two weeks. Of the 222 participants with elevated anxiety and/or depressive symptoms, 60% were male, 97% were White, and 83% were married. Their ages ranged from 24 to 77 years old; most participants held an advanced degree (master’s or above, 89%). In this occupational sample, 96% were appointed and actively serving in ministry and 98% had health insurance. Within this group of 222, 64 participants (28%) had elevated anxiety symptoms only, 46 participants (21%) had elevated depressive symptoms only, and 112 participants (51%) had both elevated anxiety and depressive symptoms.

As depicted in Table 1, out of the 222 people who had experienced elevated anxiety and/or depressive symptoms, 113 people (51%) reported currently seeing a mental health professional or having seen one in the past two years. The remaining 109 (49%) reported not having seen a mental health professional within the past two years or at any point in their lives.

**Table 1:** Mental healthcare utilization by type of elevated symptoms and timeframe

Care utilization variable	All participants (*N* = 222)	Anxiety only (*N* = 64)	Depression only (*N* = 46)	Anxiety and depression (*N* = 112)	p value
Timeframe mental health professional care received n (%)					.662
Current	85 (38.3%)	26 (40.6%)	15 (32.6%)	44 (39.3%)	
Not current but in the past 2 years	28 (12.6%)	9 (14.1%)	3 (6.5%)	16 (14.3%)	
More than 2 years ago	57 (25.7%)	14 (21.9%)	15 (32.6%)	28 (25%)	
Never	52 (23.4%)	15 (23.4%)	13 (28.3%)	24 (21.4%)	

A higher proportion of clergy with elevated anxiety symptoms only (55%), or elevated anxiety symptoms in combination with depressive symptoms (54%), were receiving or had recently received mental health services, compared to the percentage of those with depressive symptoms only (39%).

Table 2 shows the associations between selected demographic and social characteristics and service use.

**Table 2:** Demographics by mental healthcare utilization (N = 222)

Variables	Currently receiving care or had received care in the past two years (*n* = 113)	Had received care more than two years ago or never had received care (*n* = 109)	*p* value
*Demographics, n (%)*			
Gender			.054
Male (*n* = 133)	61 (46%)	72 (54%)	
Female (*n* = 88)	52 (59%)	36 (41%)	
Marital status			.010
Currently married (*n* = 181)	85 (47%)	96 (53%)	
No longer married (*n* = 15)	9 (60%)	6 (40%)	
Never married (*n* = 21)	17 (81%)	4 (19%)	
Education level (highest obtained)			.734
Bachelor's degree or lower (*n* = 25)	11 (44%)	14 (56%)	
Master's degree (*n* = 180)	94 (52%)	86 (48%)	
Doctoral degree (*n* = 15)	8 (53%)	7 (47%)	
Age range			.001
24–35 (n = 46)	31 (67%)	15 (33%)	
36–49 (n = 62)	39 (63%)	23 (37%)	
50–64 (n = 95)	35 (37%)	60 (63%)	
65–77 (n = 18)	8 (44%)	10 (56%)	
Retired status			.012
Not Retired (*n* = 202)	100 (50%)	102 (50%)	
Officially retired but currently under appointment (*n* = 8)	8 (100%)	0 (0%)	
Retired and not under appointment (*n* = 11)	4 (36%)	7 (64%)	
Rural residence status			.937
Rural (*n* = 104)	54 (52%)	50 (48%)	
Non-rural (*n* = 101)	53 (52%)	48 (48%)	
Financial stress			.005
Extremely stressful (*n* = 29)	15 (52%)	14 (48%)	
Very stressful (*n* = 38)	29 (76%)	9 (24%)	
Moderately stressful (*n* = 54)	29 (54%)	25 (46%)	
Slightly stressful (*n* = 64)	25 (39%)	39 (61%)	
Not at all stressful (*n* = 36)	15 (42%)	21 (58%)	
Depression before age of 21			< .001
Yes	50 (68%)	23 (32%)	
No	63 (42%)	86 (58%)	

We noted several significant differences in mental health service utilization by demographic and social variables (see Table 2). Clergy who were married were less likely to recently have used mental health services (*p* = 0.010). Younger (age ranges 24–35 and 36–49) clergy were more likely to have recently used mental health services than older (age ranges 50–64 and 65–77) clergy (*p* = 0.001). Compared to those who reported slight or no financial stress, clergy who reported higher levels of financial stress were more likely to use mental health services (*p* = 0.005). Those under UMC appointment (retired or not) were more likely to use services than those who were retired and not appointed (*p* = 0.012). All of the retired but appointed clergy (*n* = 8) were currently using mental health services. Female clergy were more likely to use mental health services than male clergy, with a trend toward statistical significance (*p* = 0.054). Clergy who had depression before age 21 were more likely to use services currently or in the past two years (*p* < 0.001) than clergy who did not have depression before 21 years old. Interestingly, mental health service utilization was not found to be significantly different by rural residence status (*p* = 0.937) or educational status (*p* = 0.734).

Table 3 shows the types of professionals that clergy reported going to currently or in the past two years for the care of their mental health. Participants who were seeing mental health professionals also reported the type of, if any, non-mental health professionals they were seeing within the same time period for meeting their mental health needs. Of those currently seeing a mental health professional (*n* = 85), the highest percentage of participants reported seeing a licensed professional counselor (42%), with lower percentages reporting seeing a psychologist/clinical social worker (28%) or a psychiatrist (15%), respectively. Other non-mental health professionals whom participants reported seeing include primary care physicians and spiritual directors (see table below for details).

**Table 3:** Types of professionals seen to improve or maintain mental health

	Current (*n* = 85)	Not current, in the past two years (*n* = 28)
*Type of mental health professional most frequently seen to improve or maintain mental health, n (%)*		
Psychologist or clinical social worker	24 (28.23%)	4 (14.29%)
Psychiatrist (can prescribe medications)	13 (15.29%)	1 (3.57%)
Licensed professional counselor	36 (42.35%)	20 (71.43%)
Not sure	2 (2.35%)	1 (3.57%)
Other	10 (11.76%)	1 (3.57%)
*Other types of professionals seen to improve or maintain mental health, n (%)*		
Primary care physician	52 (61.17%)	9 (33.33%)
Licensed pastoral counselor	22 (25.88%)	6 (22.22%)
Religious counselor	8 (9.41%)	3 (11.11%)
Spiritual director	10 (11.76%)	3 (11.11%)
Inner healing and/or deliverance minister	3 (3.52%)	0 (0%)
Life coach	12 (14.11%)	3 (11.11%)
Other	5 (5.88%)	1 (3.70%)
None of the above	10 (11.76%)	2 (7.41%)

The participants who indicated currently seeing non-mental health professionals reported each type of professional they saw, instead of only the type of professional that they most frequently saw. Therefore, each participant might report two or more types, such that the percentages do not add to 100%. In contrast, participants who indicated recently (in the past 2 years) using services were asked to report just the one type of non-mental health professional they had most frequently seen.

Table 4 shows the results of our exploratory analysis to see if participants’ perceived support from congregants was associated with mental health service utilization. Among the Religious Support items, the only item that significantly related to utilization was how often clergy felt loved and cared for (*p* = 0.031). Clergy who reported feeling loved and cared for by their congregants very often and never as opposed to once in a while and fairly often, were more likely to report engaging in mental health care. Therefore, the relationship between clergy perceiving being loved and cared for by their congregants and mental health service utilization is not linear, with those who reported “fairly often” being the least likely to currently use mental health services or to have used services in the past two years.

**Table 4:** Perceived support from congregants by clergy mental health service utilization timeframe

Perceived congregant support	Currently using services or had used in the past two years (*n* = 107)	Had used services more than two years ago or never used (*n* = 99)	*p* value
*How often clergy feel loved and cared for by their congregation (N = 206), n (%)*			.031
Very often	32 (68%)	15 (32%)	
Fairly often	40 (44%)	50 (54%)	
Once in a while	31 (48%)	33 (52%)	
Never	4 (80%)	1 (20%)	
*How often clergy talk to their congregation about private problems/concerns (N = 206), n (%)*			.366
Very often	7 (78%)	2 (22%)	
Fairly often	11 (50%)	11 (50%)	
Once in a while	50 (47%)	56 (53%)	
Never	39 (57%)	30 (53%)	
*Degree to which congregation would help clergy in the event of illness (N = 205), n (%)*			.404
A great deal	48 (54%)	41 (46%)	
Some	27 (44%)	34 (56%)	
A little	24 (53%)	21 (47%)	
None	7 (70%)	3 (30%)	
*Degree of comfort given to clergy if they are faced with a problem or difficult situation (N = 205), n (%)*			.548
A great deal	40 (50%)	40 (50%)	
Some	31 (47%)	35 (53%)	
A little	30 (60%)	20 (40%)	
None	5 (56%)	4 (44%)	
*Number of congregants that care about clergy as a person, and not just as clergy (N = 203), n (%)*			.837
All	3 (43%)	4 (57%)	
Most	25 (54%)	21 (46%)	
Many	34 (49%)	36 (51%)	
A couple	36 (51%)	35 (49%)	
None	6 (67%)	3 (33%)	

Table 5 depicts the associations between the social and demographic variables and three mental healthcare utilization outcomes using modified Poisson regression models. It is important to note that these outcome categories, unlike in previous tables, are not mutually exclusive.

**Table 5:** Multivariable modified Poisson regression models of mental health service utilization with sociodemographic characteristics and religious support (*N* = 201)

	Currently using services (ref = currently not using)	Currently using or have used services in the past 2 years (ref = never or used before 2 years ago)	Never used services (ref = participant has ever used services)
*Prevalence ratio [95% confidence interval];* *p value*			
Female (ref = male)	1.32 [0.91, 1.93]; .143	1.14 [0.86, 1.50]; .367	0.61 [0.34, 1.11]; .104
*Marital status (ref = married)*			
No longer married	1.04 [0.58, 1.84]; .905	1.27 [0.81, 2.00]; .292	0.49 [0.09, 2.69]; .413
Never married	1.30 [0.87, 1.94]; .206	1.15 [0.85, 1.56]; .367	0.50 [0.07, 3.62]; .491
*Retired and appointed (ref = not retired and appointed)*	3.74 [1.94, 7.20]; < .001	3.24 [2.18, 4.82]; < .001	0.01 [0.01, 0.01]; < .001
*Age (centered)*	0.99 [0.97, 1.00]; .086	0.98 [0.97, 0.99]; < .001	1.02 [1.00, 1.04]; .086
*Rural residence (ref = non-rural residence)*	0.88 [0.63, 1.25]; .479	0.93 [0.73, 1.19]; .557	1.35 [0.86, 2.12]; .192
*Financial stress*	1.63 [0.98, 2.70]; .059	1.07 [0.97, 1.18]; .196	0.46 [0.25, 0.83]; .010
*Square term of financial stress*	0.89 [0.79, 1.01]; .067	N/A	1.15 [0.98, 1.35]; .081
*Depression before the age of 21*	1.91 [1.34, 2.70]; < .001	1.50 [1.16, 1.94]; .002	0.38 [0.17, 0.83]; .016
*Frequency of loved and cared for by congregants (ref = fairly often)*			
Once in a while or never	1.06 [0.70, 1.60]; .789	1.03 [0.75, 1.42]; .837	0.88 [0.51, 1.50]; .633
Very often	1.34 [0.89, 2.02]; .156	1.38 [1.03, 1.86]; .033	0.82 [0.43, 1.56]; .537
*Education level (ref = Master's)*			
Bachelor’s or lower	0.84 [0.46, 1.55]; .583	0.87 [0.56, 1.35]; .540	1.59 [0.80, 3.17]; .186
Doctoral	1.31 [0.75, 2.30]; .341	1.25 [0.79, 1.97]; .334	0.37 [0.06, 2.13]; .266
Constant	0.15 [0.09, 0.27]; < .001	0.30 [0.22, 0.42]; < .001	0.64 [0.41, 1.00]; .048

N/A indicates that the square term, when included in the model, wasn’t significantly associated with the outcome. Therefore, the square term is not included in the final model specification of this particular outcome.

Participants who were retired were statistically significantly more likely to have used mental health services across all three timeframe outcomes as compared to non-retired clergy (prevalence ratio 3.74, 95% CI 1.94–7.20; *p* < 0.001; prevalence ratio 3.24, 95% CI 2.18–4.82; *p* < 0.001; prevalence ratio 0.01, 95% CI 0.01–0.01; *p* < 0.001, respectively). Participants with greater financial stress were significantly less likely to have never used mental health services (prevalence ratio 0.46, 95% CI 0.25–0.83; *p* = 0.010). Participants who reported experiencing depression before the age of 21 were significantly more likely to use services, across all three timeframe outcomes, than those who did not experience depression before 21 years old (prevalence ratio 1.91, 95% CI 1.34- 2.70; *p* < 0.001; prevalence ratio 1.50, 95% CI 1.16–1.94; *p* = 0.002; prevalence ratio 0.38, 95% CI 0.17–0.83; *p* = 0.016, respectively).

Compared to clergy who reported feeling loved and cared for by congregants fairly often, clergy who reported feeling loved and cared for very often were significantly more likely to have used mental health services currently or in the past 2 years (prevalence ratio 1.38, 95% CI 1.03–1.86; *p* = 0.033). However, no significant difference was found between participants who reported feeling loved and cared for by their congregants never or once in a while vs fairly often. Older age was significantly associated with being less likely to receive care in the past two years (prevalence ratio 0.98, 95% CI 0.97–0.99; *p* < 0.001).

Gender, marital status, urban/rural residence, and level of education were not associated with service use in the controlled analyses.

## Discussion

Our findings indicate that almost half (49.1%) of clergy with recent depressive and/or anxiety symptoms did not get the professional services that they could have benefited from in the past two years. While we could not find an exact comparison with a two-year timeframe, studies of people in the USA with mental illness (depression, anxiety, and other disorders) indicate that 55.2% (National Alliance on Mental Illness, 2019) and 56.4% (Mental Health in America, 2019) did not use mental health care in the past year. Thus, United Methodist clergy in North Carolina are on par with Americans overall in utilizing mental health services. Nevertheless, a gap of approximately half of clergy not utilizing services they could benefit from is concerning. Getting mental health support could help clergy flourish as individuals and help them serve their congregants better. Additionally, they would be more knowledgeable about available services and referrals if they had sought help themselves and further could serve as role models on seeking and receiving services.

We asked participants which kind of providers they saw. Clergy reported that their current primary providers of mental health services were primary care physicians (61%), followed by licensed professional counselors (LPC) (42%), psychologists/clinical social workers (28%), and licensed pastoral counselors (26%). It is important to educate primary care physicians, psychologists, LPCs, and social workers on the experiences of clergy, including their stressors and tendency to overextend themselves because they are called to their vocation (Proeschold-Bell & Byassee, 2018), so they can provide optimal care.

Perceptions of being loved and cared for by one’s congregants had a nonlinear association with using mental health care in this study. Clergy who reported receiving support from their congregation very often, as opposed to fairly often, were more likely to report engaging in mental health therapy. While our measure may be too crude to describe the exact relationship between congregant support for clergy and clergy mental health service use, these findings indicate that such a relationship exists. It may be that clergy who have felt very supported are encouraged to seek the care that they need and feel able to risk experiencing mental health stigma. Those who never feel supported might feel like their need for help is so high that they are willing to risk being stigmatized. Qualitative research may be the best next step to understand the interplay of underlying factors, which may include stigma, support, and work expectations.

Clergy who reported greater financial stress were less likely to never have received services; previous research indicates a positive association between financial stress and persistent moderate and severe depressive symptoms among clergy (Hybels et al., 2018), making it fortunate that financially stressed clergy are more likely to have sought mental health care at some point in their lives. While low income in non-clergy populations has been a barrier to seeking mental health services (Magaard et al., 2017), nearly all of the current study’s population of United Methodist clergy had health insurance, which may have facilitated their mental healthcare use. Clergy of other faiths and denominations may be less likely to have health insurance, and if so, their rates of mental healthcare seeking may be lower than those reported here.

In the current study, clergy gender was not associated with service use which is not consistent with findings from a national survey of Americans that reported higher mental service utilization for females (51.2%) than males (37.4%) (Substance Abuse and Mental Health Services Administration, 2020).

Older age was significantly associated with being less likely to receive care within the past two years vs never or more than two years ago. This might be explained by the younger generation viewing mental health distress, such as depression, with less stigma. There has been a significant decrease in public stigma in the USA, age being one of the contributing factors (Pescosolido et al., 2021).

Not surprisingly, clergy with a history of depression were more likely to have ever used mental health services as depression can be viewed as a chronic disease (Rakel, 1999). Retired clergy were more likely to use mental health services; this could be due to the increased chance of depression related to loneliness and social isolation often associated with retirement (Alpass & Neville, 2003).

More should be done to encourage mental health service utilization and bridge the treatment gap among the clergy with elevated anxiety and/or depressive symptoms who are not receiving mental health care. Clergy with current unmet mental health needs should be encouraged to seek mental health care and barriers such as stigma should be addressed (Parcesepe & Cabassa, 2013). There are not easy solutions to facilitate needed mental healthcare use among people who are not in care. This study suggests that, for United Methodist clergy special, attention should be paid to clergy who are older (ages 50–77) and who do not feel loved and cared for by congregants. We suggest that, as a way to destigmatize care, supervisors could help monitor clergy’s needs and normalize discussions regarding mental health services. Denominational officials could make mental healthcare programs available and affordable. Among clergy in the current study, mental health co-pays ranged from $25-$60 per session and one conference (roughly 40% of this study’s participants) offered reimbursement for co-pays for clergy completing an application. We did not find evidence that supports that clergy in this context experience financial stress that prohibits them from accessing mental health services. Our findings hint that perhaps more could be done to decrease the treatment gap reported here among United Methodist clergy, and yet many clergy in other denominations have even fewer resources available.

In addition to professional mental health services and discussion platforms, clergy may benefit from peer support and enhanced social networks to improve and maintain their mental health (Lutz & Eagle, 2019). Among online communities of people with serious mental illness, Naslund and colleagues (2016) showed evidence that peer-to-peer support could promote mental health-seeking behavior by helping overcome stigma and empowering persons living with mental conditions. Peer support has also been found to be acceptable and feasible and lead to future use of formal mental health services among a variety of professionals who care for others and may otherwise resist seeking care for themselves (e.g., child protection workers, caregivers for people with dementia, police officers, veterans, and mothers caring for children with special needs) (Evans et al., 2020). Similarly, Miles and Proeschold-Bell (2012) found that clergy who stayed in unfacilitated peer support groups for two years indicated less psychological distress compared to themselves two years earlier; however, clergy who dropped out of peer support groups did not get worse, indicating that peer support might not work for all and that clergy may be aware when a group is not helping them. Therefore, while acknowledging the potential benefits of peer support groups, we must also highlight the need to tailor them to individual clergy.

In our study, clergy not only reported turning to professional mental health providers for help, but also reported seeking services from a spiritual director in order to improve/maintain their mental health (12%). Given the beneficial relationship of social networks and reduced depressive symptoms among clergy (Lutz & Eagle, 2019), future studies could examine clergy’s use of spiritual directors or other non-mental health professionals in their network to explore the potential benefits.

Previous reports have clarified the mental health treatment gap by considering different groups of people with unmet need, for example, those who have access to treatment but do not utilize it (Mental Health in America, 2019). Further studies, especially qualitative ones, should look into the root causes of the gap in clergy mental health care in order to promote care utilization. In the current study, licensed professional counselors (LPCs) seem to be the preferred provider of clergy utilizing care. Future studies may be aimed at determining whether LPCs feel the need for more education on how to support clergy. Furthermore, to try to decrease the mental healthcare gap, efforts could be made to engage psychologists, psychiatrists, and social workers in reaching out to clergy and making it known that they will be welcoming and provide good mental health care to people of strong faith. Two-way communication between clergy and mental health professionals about how to collaborate on the mental health care of congregants is recommended (Rudolfsson & Milstein, 2019); the current study indicates that the same is needed for the mental health care of clergy themselves.

### Study Strengths and Limitations

The study’s limitations include that depressive and anxiety symptoms were self-reported and not verified by a psychologist or other provider capable of providing a diagnosis and that the data are cross-sectional and causal relationships cannot be inferred. Further, the sample is composed of clergy from one Christian denomination, limiting the study’s generalizability, although some studies of clergy indicate that clergy across denominations engage in similar activities that involve stress (DeShon, 2010). Strengths of the study include the high response rate and the broad range of mental health service utilization questions asked, which included assessing timing of most recent mental health care.

## Conclusion

Given that other studies report above-average levels of depressive and anxiety symptoms among clergy, particularly United Methodist clergy (Proeschold-Bell et al., 2013), the current study’s findings are heartening in that rates of United Methodist clergy mental health service use are on par with those of other Americans. Nevertheless, more could to be done to address the 49% gap in clergy with elevated symptoms who are not utilizing professional mental health care. Clergy serve their community and impact the lives of their congregants. If clergy themselves are feeling distressed and not seeking services they can benefit from, it may affect their service to others. To that end, it is important to support clergy, not only to improve their own wellbeing, but also for them to maximally support others who are in their sphere of influence.

## Data Availability

The datasets generated during the current study are not publicly available, but de-identified data will be made available on reasonable request where such requests are compliant with receipt of ethical approval from the sending and receiving hosts’ institutional ethics review boards.
